# Ensemble model for predicting chronic non-communicable diseases using Latin square extraction and fuzzy-artificial neural networks from 2013 to 2019

**DOI:** 10.1016/j.heliyon.2023.e22561

**Published:** 2023-11-20

**Authors:** Nevena Rankovic, Dragica Rankovic, Igor Lukic, Nikola Savic, Verica Jovanovic

**Affiliations:** a Department of Cognitive Science and Artificial Intelligence, Tilburg School of Humanities and Digital Sciences, Tilburg University, Warandelaan 2, Tilburg, 5037 AB, Netherlands; b Department of Mathematics, Statistics and Informatics, Faculty of Applied Sciences, Union University “Nikola Tesla”, Dusana Popovica 22, Nis, 18000, Serbia; c Department of Preventive Medicine, Faculty of Medical Sciences, University of Kragujevac, Svetozara Markovica 69, Kragujevac, 34000, Serbia; d Faculty of Business Valjevo, Singidunum University, Zeleznicka 5, Valjevo, 14000, Serbia; e Institute of the Public Health “Dr. Milan Jovanovic Batut”, dr Subotica starijeg 5, Belgrade, 11000, Serbia

**Keywords:** Chronic non-communicable disease, Decision tree, Support vector regressor, ANN-L36-fuzzy model, Orthogonal vector plans

## Abstract

**Background:**

The presented study tracks the increase or decrease in the prevalence of seventeen different chronic non-communicable diseases in Serbia. This analysis considers factors such as region, age, and gender and is based on data from two national cross-sectional studies conducted in 2013 and 2019. The research aims to accurately identify the regions with the highest percentage of affected individuals, as well as their respective age and gender groups. The ultimate goal is to facilitate organized, free preventive screenings for these population categories within a very short time-frame in the future.

**Materials and methods:**

The study analyzed two cross-sectional studies conducted between 2013 and 2019, using data obtained from the Institute of Public Health of Serbia. Both studies involved a total of 27801 participants. The study compared the performance of Decision Tree and Support Vector Regressor models with artificial neural network (ANN) models that employed two encoding functions. The new methodology for the ANN-L36 model was based on artificial neural networks constructed using a Latin square (L36) design, incorporating Taguchi's robust design optimization.

**Results:**

The results of the analysis from three different models have shown that cardiovascular diseases are the most prevalent illnesses among the population in Serbia, with hypertension as the leading condition in all regions, particularly among individuals aged 64 to 75 years, and more prevalent among females. In 2019, there was a decrease in the percentage of the leading disease, hypertension, compared to 2013, with a decrease from 34.0% to 32.2%. The ANN-L36 model with Fuzzy encoding function demonstrated the highest precision, achieving the smallest relative error of 0.1%.

**Conclusion:**

To date, no studies have been conducted at the national level in Serbia to comprehensively track and identify chronic diseases in the manner proposed by this study. The model presented in this research will be implemented in practice and is set to significantly contribute to the future healthcare framework in Serbia, shaping and advancing the approach towards addressing these conditions. Furthermore, experimental evidence has shown that Taguchi's optimization approach yields the best results for identifying various chronic non-communicable diseases.

## 1 Introduction

The World Health Organization (WHO) [1] reported in 2014 that chronic non-communicable diseases caused 68% of all deaths worldwide in 2012, with cardiovascular diseases being the main cause (46.2%). Of those who died from these diseases, 42% were under the age of 70, and low- and middle-income nations accounted for the majority of these deaths (82%) and premature deaths. According to estimates, mortality rates from diabetes, cancer, chronic respiratory conditions, and cardiovascular diseases will rise by 2030, with developing and impoverished nations bearing the brunt of this increase. In Europe, chronic non-communicable diseases caused 77% of the disease burden and 86% of mortality, with leading causes including ischemic heart disease, cerebrovascular diseases, depression, back and neck pain, and lung cancer. Ischemic heart disease, cerebrovascular diseases, lung cancer, liver cirrhosis, and self-harm were among the leading causes of premature death [1], [2].

Serbia is experiencing a rise in the prevalence of chronic non-communicable diseases, much like other nations. Diabetes, unipolar depression, lung cancer, ischemic heart disease, and cerebrovascular disease are among the leading causes of mortality in the nation [3], [4]. While heart and vascular disorders have seen a decrease in mortality, diabetes, malignant tumors, and obstructive lung disease have shown an increase in mortality [5]. Additionally, the prevalence of chronic illnesses such as high blood lipid levels, diabetes, depression, hypertension, and allergies has increased among adults. These trends can be explained by the aging process of the population, as well as socioeconomic and environmental factors that influence health in the country. Chronic non-communicable diseases represent a serious public health issue that requires effective measures of prevention, diagnosis, treatment, and rehabilitation, considering their long-term impact and negative consequences for individuals, families, and society [6], [1].

The significance and justification for utilizing analyses in public health research become evident through studies that provide insight into the mechanisms by which determinants of health influence health outcomes. For instance, trends in the incidence rates of cardiovascular diseases demonstrate that the risk of their occurrence increases with age [7]. Among adult males, these diseases gradually increase from their forties to their sixties, while in females, this trend begins from their fifties. Ischemic heart disease is estimated to be 2-5 times more common in middle-aged males compared to females [8]. Atherosclerosis is the fundamental cause of cardiovascular diseases, although the exact reasons for its development are still unknown. However, it is known that there are factors contributing to its onset at a younger age [9]. Malignant tumors generally increase with age, with certain sites such as bladder carcinoma, lymphoma, leukemia, and liver cancer being more prevalent in males than females [8]. Chronic obstructive pulmonary disease is most commonly diagnosed in older individuals, while the prevalence of type 2 diabetes increases with age in both genders [10]. According to the literature, the association between chronic non-communicable diseases and age is stronger than the association with any other individual characteristic. It is so strong that differences in disease occurrence among population subgroups defined based on other characteristics often cannot be explained without considering possible variations in age structure. However, when it comes to chronic non-communicable diseases, it is challenging to differentiate how much of their occurrence is due to aging processes and pre-existing degenerative tissue changes, which increase susceptibility to factors, as opposed to the effects of exposure to and/or accumulation of factors influencing the onset of specific diseases or a prolonged latent period [8].

For instance, systolic blood pressure is such a case. While most studies indicate that systolic blood pressure increases with age, research conducted on several isolated populations did not show such a trend [11]. This suggests that the rise in systolic blood pressure with age is not merely a physiological phenomenon but is associated with changes in lifestyle occurring with the aging process in urban populations. These changes are linked to increased body weight and reduced physical activity.

Similarly, the increased incidence of lung cancer and liver cirrhosis in older age groups can, at least partially, be explained by their correlation with risky lifestyles. Studies have shown that both men and women are equally affected by chronic obstructive pulmonary disease (COPD), contradicting previous findings that had suggested the disease was more common in men. This shift can be attributed to the growing prevalence of smoking among women [8].

The surveillance of chronic non-communicable diseases (NCDs) and analysis of their prevalence serve multiple important purposes and objectives. These include timely health policy planning, identification of trends and risks, development of effective prevention strategies, early detection, and treatment. Such data are crucial for informed health policy planning and resource allocation based on priorities. Surveillance enables prevention and early diagnosis, improves patient outcomes, and reduces the burden of disease with global impact. The evaluation of intervention effectiveness allows for assessing outcomes and identifying areas for improvement. Moreover, it provides relevant information for raising awareness and public education, promoting healthy lifestyles, and supporting informed health decision-making. Through the analysis of disease prevalence and trends, the effectiveness of measures can be determined, and areas for further enhancement of strategies can be identified. The surveillance of chronic non-communicable diseases has a comprehensive impact on population health and well-being.

In today's society, the prevalence of numerous stress-inducing factors contributes to the emergence and progression of diseases. Hence, alongside lifestyle modifications, regular preventive and diagnostic check-ups are crucial, and healthcare systems need to be enhanced with intelligent techniques. The healthcare industry incessantly seeks novel approaches to enhance the quality of healthcare services and improve the treatment outcomes for complex diseases. One area showing promise is Artificial Intelligence (AI), encompassing various branches such as Machine Learning (ML) algorithms and Artificial Neural Networks [12], [13]. These technologies offer diverse opportunities for healthcare improvement, ranging from disease outbreak prediction and monitoring to identifying high-risk patients and devising personalized treatment plans. ML can aid healthcare by shifting the focus from reactive to preventive measures. By leveraging a patient's medical history and other pertinent data, ML algorithms can provide treatment recommendations, enabling the identification of potential health risks before they escalate. For instance, a machine learning model can assess a patient's genetic composition and medical history to detect early risk factors for heart disease, facilitating proactive interventions to avert life-threatening conditions. Through the analysis of extensive data-sets, ML algorithms and ANNs can discern patterns and correlations that may elude human researchers [13]. This knowledge can inform the development of more effective disease prevention and management strategies, such as targeted vaccination campaigns or early warning systems for outbreaks. Moreover, AI can be instrumental in identifying gaps in healthcare provision and hidden risk categories. By scrutinizing patient data, ML algorithms can identify high-risk individuals who might be overlooked by traditional risk assessment tools [14]. This information can be used to formulate improved care plans, ensuring that high-risk patients receive adequate attention to effectively manage their conditions [15], [16]. In conclusion, the integration of AI in healthcare holds immense potential to revolutionize the industry by enhancing the quality of healthcare services, improving the treatment outcomes for complex diseases, and revealing previously unidentified gaps in healthcare provision and hidden risk categories. As these technologies advance and evolve, it is expected that innovative applications of AI in healthcare will continue to emerge in the forthcoming years [14], [15], [16].

The creation of an innovative methodology that makes use of the experiment's sturdy construction and incorporates Taguchi's orthogonal vector plans highlights the novelty of this research [17]. This unique approach aims to streamline the training, testing, and validation phases of artificial neural networks, significantly reducing the number of iterations required and consequently minimizing estimation time, particularly in diagnostic applications.

One key advantage of this design construction lies in its ability to render the process insensitive to variation, while simultaneously ensuring that factors are neither lost nor duplicated [18]. This feature is made possible through Taguchi's hyper-parameter optimization, enabling high-quality estimation that accounts for all uncontrolled factors throughout the entire operating phase, accompanied by reliable certainty quantification.

It is crucial to note that the success of any chosen AI tool often hinges upon accurate predictions during the design stage. By leveraging Taguchi's orthogonal vector plans, the constructed ANN model holds the potential to generate realistic predictions for an extensive range of *N-of-1* study designs, diverse treatment profiles, and varying patient characteristics, establishing its versatility and wide-ranging applicability.

The main research objectives that defined our study are as follows:

***RO1***: Introducing the new methodology with two coding functions for the ANN-L36 model for predicting the increase/decrease or constant dissemination of chronic non-communicable diseases from 2013 to 2019.
***RO2***: Comparison of the new ANN-L36 model with ML algorithms and other ANNs based on different hidden layer functions.

The remainder of the document is structured as follows: In Section 2, the previously published research employing various AI tools in medical diagnostics is reviewed; in Section 3, the new ANN-L36 model is proposed; in Section 4, the results are shown; in Section 5, the results are discussed and the answers to the research objectives are provided; and in Section 6, concluding remarks are provided.

## 2 Related work

In this section, we will describe recent studies relevant to our research and present the authors' objectives and approaches for addressing medical data-related challenges. Specifically, we will discuss research conducted in the field of enhancing medical diagnosis and making various health predictions using various ML algorithms and ANNs. Using genetic algorithms, decision trees, and synthetic minority oversampling approaches, the authors of the research [19] created a unique predictive model, called PMSGD, to identify diabetes mellitus in the Pima Indians Diabetes Database (PIDD) dataset. Using fuzzy logic and decision trees, Johnson et al. [20] reached an 88% accuracy rate in identifying heart disease in another study.

Two models were provided in a study published in [21]: a Random Forest, Decision Tree and a probabilistic neural network based on dynamic damping fitting. The natural verbal complaints made by patients were used as user-generated data in these models. Results from different categorization algorithms such as Decision Trees, Random Forest Trees, and Extra Trees that obtained accuracy rates of 98%, 99%, and 93%, respectively, were presented in the study in [22].

Support Vector Machine (SVM) is widely recognized for its high classification accuracy in pattern classification. Researchers in [23] utilized SVM to create a Chaotic Multi-Swarm Whale Optimizer, enhancing medical diagnostics. SVM has also proven effective in brain tumor segmentation and classifying different types of gliomas, as demonstrated in studies from [24] and [25]. A succinct three-step method was suggested by researchers in [26] to enhance the interpretability of EEG-SVM experiments. This approach emphasizes the important parts of developing a model, such as cross-validation, hyperparameter tuning, and normalization. It emphasizes how crucial a role these three components play in determining how well SVM models diagnose and predict a range of neurological and mental diseases. In [27], the adaptive boost Least Square (LS)-SVM classification approach was employed for epileptic seizure diagnosis applications based on time-series signal classification. Additionally, for medical diagnostic information systems, a study [28] suggested integrating SVM with a metaheuristic salp swarm algorithm.

An automated medical decision support system using a convolutional neural network (CNN) or EfficientNet implementation and ten layers of stratified cross-validation was one of the main goals of a study published in [29]. A Heterogeneous Modified Artificial Neural Network (HMANN) for the Internet of Medical Things (IoMT) platform was introduced in [30], another study, for the purpose of early detection, segmentation, and diagnosis of chronic renal failure. In a research published in [31], multilayer perceptron neural networks (MLP) and convolutional neural networks (CNN) were employed to detect breast cancers in order to aid in the early diagnosis of breast cell malignancies. The authors of [32] examined a number of CNNs that are often used in medical imaging, including Fully-CNN, AlexNet, GoogleNet, ResNet, and Region-based CNN. SVM is a popular pattern classification technique in medical diagnosis because of its high classification accuracy and other demonstrated qualities. Furthermore, it has been demonstrated that integrating SVM with other algorithms like the adaptive boost LS-SVM classification technique, salp swarm algorithm, and chaotic multi-swarm whale optimizer works well in medical diagnostic information systems. Automated medical decision support systems have been suggested to use CNN and EfficientNet implementations. Finally, early identification, segmentation, and diagnosis of brain tumors, breast cancer, and chronic renal failure have been achieved by the use of HMANN, MLP, and CNN.

The researchers in [33] conducted a review that substantiates its points by presenting two specific case studies, which exemplify the utilization of AI for predicting epileptic seizure occurrences and addressing issues related to a dysfunctional urinary bladder. Their study's conclusions highlighted the possibility of accurately identifying seizure sinks and generators by applying a time-varying variant of the spectrum-weighted adaptive directed transfer function (swADTF). After that, a multilayer perceptron used the extracted attributes as classification inputs. It was also proposed to develop a novel fitness function that is insensitive to skewed data distributions. An average sensitivity of 84.82% at a time-in-warning of 10% was observed on the held-out dataset, indicating an improvement above previous seizure prediction performances. Each of the three bispectrum-extracted features significantly distinguished between the interictal and preictal stages (p<0.05), according to their preliminary research. Test accuracy of 73.26%, 72.64%, and 78.11% were obtained for the mean of magnitudes, normalized bispectral entropy, and normalized squared entropy, respectively. Based on effective connectivity measures, electrode selection methods are suggested for future investigations in seizure forecasting.

The primary aims of the study [34] were twofold: (1) to use algorithms to find combinations of clinical traits linked to COVID-19 that can predict patient outcomes, and (2) to create an AI-powered tool that can identify people who are at risk of presenting with a more severe illness at first. The severe COVID-19 consequence known as acute respiratory distress syndrome (ARDS) was predicted by the predictive models by training on historical data. Findings based on data collected from two hospitals in Wenzhou, Zhejiang, China revealed that mildly elevated alanine aminotransferase (ALT), the presence of myalgias, and elevated hemoglobin levels were the most predictive clinical features during initial presentation for later development of ARDS. The developed predictive models achieved an accuracy ranging from 70% to 80% in accurately predicting severe cases, leveraging the insights gleaned from the historical patient data of these two hospitals.

A novel method for the prediction of five common chronic diseases such as breast cancer, diabetes, heart attacks, hepatitis, and kidney disease combining augmented artificial intelligence, specifically an Artificial Neural Network with Particle Swarm Optimization (PSO) is presented in the study [35]. The suggested model's prediction capabilities are evaluated by comparing its performance with that of seven other categorization methods. Attaining an accuracy of 99.67%, the ANN prediction model, built with a PSO-based feature extraction strategy, outperforms other cutting-edge classification techniques. Notably, it is discovered that the characteristics of the data used for classification have an impact on the classification model's accuracy. When benchmarked against other chronic disease datasets, the study's results perform better than those of other benchmark methodologies. Furthermore, the streamlined ANN processing takes less time than other approaches including support vector machine-based, random forest, and deep learning techniques. The results of this study could be used to develop online diagnosis tools and to diagnose chronic illnesses early in hospital settings.

Using AI and big data machine learning approaches, the researchers in [36] created a new predictive model for diabetic kidney diseases (DKD). Through the examination of 64059 diabetes patients' electronic medical records, artificial intelligence was able to detect time series patterns linked to the worsening of DKD over a six-month duration. The constructed predictive model achieved 71% accuracy in predicting DKD aggravation and demonstrated a significant correlation between DKD aggravation and a higher incidence of hemodialysis over a 10-year period, suggesting the potential for more effective intervention strategies based on early detection of DKD progression.

In the study [37], the efficacy of several machine learning algorithms in predicting unfavorable outcomes in patients with inflammatory bowel disease (IBD) was evaluated by establishing a training model cohort and validating it using an additional cohort. The relative performances of the various models such as LASSO and Ridge regressions, Support Vector Machines, Random Forests, and Neural Networks were examined, and the models with the strongest predictors were presented. The OptumLabs® Data Warehouse provided de-identified administrative claims and electronic health record (EHR) data from 72178 training patients and 69,165 validation patients, which were used in the study. Notably, the Random Forest and LASSO models demonstrated high accuracies (AUCs 0.70-0.92), while the artificial neural network also performed well in most of the models (AUCs 0.61-0.90). These results demonstrate the possibility for risk assessment and the deployment of preventative treatments in clinical settings by employing sophisticated AI models on extensive longitudinal datasets to forecast unfavorable outcomes in IBD patients.

According to available literature and current understanding, no comparable studies have used ANN designs based on Taguchi's Orthogonal vector plans using two coding functions to predict chronic non-communicable diseases, despite the growing body of research in computer-aided medical diagnostics. Moreover, the accuracy found in this study is superior to that of any other scientific study. Furthermore, there are no similar studies using the most straightforward ANN constructed in this way, with a high convergence rate (less than eight iterations are required to perform the experiment).

To conclude, our goal is to explore which of the proposed combination of six distinct methodologies for the artificial neural network ANN-L36 gives the best results within the realm of medical diagnostics for chronic communicable diseases [38], [39], [40]. This involves:

• Analyzing the acquired Mean Magnitude of Relative Error (MMRE) values.
• Investigating various activation functions for the hidden layer of the ANN.
• Identifying the most effective techniques for encoding and decoding input values, including approaches like the fuzzification/defuzzification methods.
• Minimizing the number of necessary experiments while ensuring robust outcomes.
• Conducting thorough testing and validation of the model across diverse datasets pertinent to chronic non-communicable diseases.

## 3 Methodology remarks

To accomplish the primary research objectives, we will outline the following in this section: ML algorithms such as Decision Tree and Support Vector Machine, and ANN with Radial Basis Function in standardized and normalized way will be showcased in subsection 3.1, and new methodology for robust design of the experiment for the ANN-L36 model constructed according to Taguchi's Orthogonal vector plans will be given in subsection 3.2, along with dataset description in subsection 3.3.

### 3.1 Different ML algorithms and RBF ANNs

Decision tree classification is a rapid and effective method for categorizing datasets. The process involves two stages: training the classifiers with features from the proposed dataset and then testing their performance, typically measured in terms of accuracy. Creating sparse and dense regions in the data space is the fundamental principle behind decision trees. As long as the data isn't sufficiently homogeneous, the tree stops. After training, a decision tree capable of generating well-categorized predictions is returned [41], [42]. In medical diagnostics, this algorithm can be referred to as a method of ‘*coming to diagnosis*’, essentially classifying a patient's condition. Parameters such as max depth, min samples split, min samples leaf, max features, and min impurity decrease are used to fine-tune the algorithm. To explore the incidence rate of chronic non-communicable diseases by region, age group, and gender for the identified ‘leader disease’, the gain ratio formula, a variation on the information gain formula that reduces bias, is a better choice in this case.

The underlying concept beneath the SVM algorithm is the development of an optimum hyper-plane that can be used to classify patterns that are linearly separable. The ideal hyperplane is one that is selected from the collection of hyper-planes for pattern classification in a way that optimizes the margin or the distance between the hyperplane and the pattern's closest point. This algorithm's primary objective is to maximize the margin in order to enable accurate pattern classification. The better the pattern classification, the larger the margin. In the case of non-linear separable patterns, the supplied pattern is mapped into a new space, often one with a higher dimension, where it becomes linearly separable. The kernel function, Φ(x) can be used to map the given pattern into space with more dimensions, i.e., x → Φ(x). Adopting an alternative kernel function is essential for the SVM-based classification; common ones are linear, poly, rbf, and sigmoid [43], [44]. While SVM is frequently used in classification problems, Support Vector Regressor (SVR) algorithm is utilized for regression models. SVR has numerous advantages including as the capacity to manage high-dimensional data, deal with outliers skillfully, and deliver reliable results even with a small number of training samples. Its versatility and effectiveness make SVR a valuable tool for regression analysis in diverse research fields. Support Vector Regressor (SVR) predictive analysis is used to evaluate the influence of chronic non-communicable diseases on residents' health condition depending on a number of factors.

The Radial Basis Function Artificial Neural Network (RBF ANN) is distinct from other ANN topologies because it uses a single hidden layer and computes the output in a different way. The hidden layer of an RBF ANN projects data into a higher-dimensional space, and its construction is based on the cover theorem. Consequently, there should be more neurons in the hidden layer than there are in the input layer. Either a linear activation function or no activation function at all may be used in the output layer. Different kinds of classifiers are frequently used for comparison. In this study, we compare RBF neural network classifiers trained with the symmetric Fuzzy Means algorithm and standard Support Vector Machine classifiers with Gaussian kernel functions [45].

### 3.2 Experimental setup - ANN-L36

**Figure 1:**
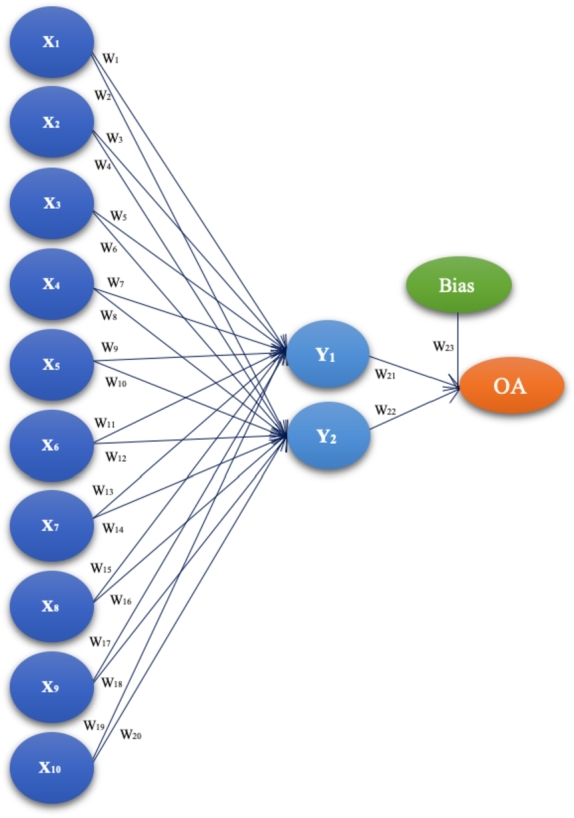
ANN-L36 constructed based on Taguchi's orthogonal vector plan L36 (2^11^3^12^).

We will introduce a novel ANN-based methodology in this subsection that was built using Taguchi's orthogonal vector plan. The number of parameters, weighting coefficients, and levels of each parameter determine Taguchi's robust experimental design in each orthogonal plane. When organizing as many tests as feasible in which all conceivable discrete values of each input component are combined, there are several strategies for establishing the dependence of the output on the input quantities using the FFP (*Full Factorial Plan*) [46], [47]. The number of experiments needed is L^*P*^, where L is the number of levels of factor variation and P is the number of factors, when we have a big number of input factors (more than 6) and at a large number of levels (greater than 5) [48], [49]. In other words, how many times should each level be tested for each parameter? A full factorial design necessitates performing N = 2^11^3^12^ = 1 088 391 168 experiments. For instance, when utilizing three levels with 12 parameters and two levels with 11 parameters, the number of iterations needed is N = L^*P*^. Using a Taguchi orthogonal plan with 12 parameters (weight coefficients) at three levels and 11 parameters at two levels, only OA|36,2^11^,3^12^|= 36 experiments are needed. OA is the commonly used acronym to refer to Orthogonal Array in Taguchi's optimization method. In this research, OA will represent OA(ANN_*i*_), which signifies the actual risk calculated using the ANN-L36 orthogonal vector plan derived from *Latin square* extraction. Taguchi's robust design method reduces the number of experiments by 99.9999999669% (0.999999999669 = 1-(36/1 088 391 168)). Taguchi's orthogonal vector plan considers all parameters equally while taking a chosen subset of combinations and eliminating duplication. Additionally, they can be assessed separately from one another. Every level of a given parameter is observed to have an orthogonal vector plan. Every one of the (*P-1*) additional parameters is evaluated at least once at all L levels. The proposed ANN architecture in our experiment is ANN-L36. This architecture is based on the Taguchi orthogonal array L36 with 11 parameters on two levels L1 and L2, and 12 parameters on three levels L1, L2, and L3, where W_*i*_, i = 1, …,12 Fig. 1, Table 1.

**Table 1:** Taguchi's orthogonal vector plan L36 (2^11^3^12^).

**ANN-L36**	W_1_	W_2_	W_3_	W_4_	W_5_	W_6_	W_7_	W_8_	W_9_	W_10_	W_11_	W_12_	W_13_	W_14_	W_15_	W_16_	W_17_	W_18_	W_19_	W_20_	W_21_	W_22_	W_23_
ANN1	L1	L1	L1	L1	L1	L1	L1	L1	L1	L1	L1	L1	L1	L1	L1	L1	L1	L1	L1	L1	L1	L1	L1
ANN2	L1	L1	L1	L1	L1	L1	L1	L1	L1	L1	L1	L2	L2	L2	L2	L2	L2	L2	L2	L2	L2	L2	L2
ANN3	L1	L1	L1	L1	L1	L1	L1	L1	L1	L1	L1	L3	L3	L3	L3	L3	L3	L3	L3	L3	L3	L3	L3
ANN4	L1	L1	L1	L1	L1	L2	L2	L2	L2	L2	L2	L1	L1	L1	L1	L2	L2	L2	L2	L3	L3	L3	L3
ANN5	L1	L1	L1	L1	L1	L2	L2	L2	L2	L2	L2	L2	L2	L2	L2	L3	L3	L3	L3	L1	L1	L1	L1
ANN6	L1	L1	L1	L1	L1	L2	L2	L2	L2	L2	L2	L3	L3	L3	L3	L1	L1	L1	L1	L2	L2	L2	L2
ANN7	L1	L1	L2	L2	L2	L1	L1	L1	L2	L2	L2	L1	L1	L2	L3	L1	L2	L3	L3	L1	L2	L2	L3
ANN8	L1	L1	L2	L2	L2	L1	L1	L1	L2	L2	L2	L2	L2	L3	L1	L2	L3	L1	L1	L2	L3	L3	L1
ANN9	L1	L1	L2	L2	L2	L1	L1	L1	L2	L2	L2	L3	L3	L1	L2	L3	L1	L2	L2	L3	L1	L1	L2
ANN10	L1	L2	L1	L2	L2	L1	L2	L2	L1	L1	L2	L1	L1	L3	L2	L1	L3	L2	L3	L2	L1	L3	L2
ANN11	L1	L2	L1	L2	L2	L1	L2	L2	L1	L1	L2	L2	L2	L1	L3	L2	L1	L3	L1	L3	L2	L1	L3
ANN12	L1	L2	L1	L2	L2	L1	L2	L2	L1	L1	L2	L3	L3	L2	L1	L3	L2	L1	L2	L1	L3	L2	L1
ANN13	L1	L2	L2	L1	L2	L2	L1	L2	L1	L2	L1	L1	L2	L3	L1	L3	L2	L1	L3	L3	L2	L1	L2
ANN14	L1	L2	L2	L1	L2	L2	L1	L2	L1	L2	L1	L2	L3	L1	L2	L1	L3	L2	L1	L1	L3	L2	L3
ANN15	L1	L2	L2	L1	L2	L2	L1	L2	L1	L2	L1	L3	L1	L2	L3	L2	L1	L3	L2	L2	L1	L3	L1
ANN16	L1	L2	L2	L2	L1	L2	L2	L1	L2	L1	L1	L1	L2	L3	L2	L1	L1	L3	L2	L3	L3	L2	L1
ANN17	L1	L2	L2	L2	L1	L2	L2	L1	L2	L1	L1	L2	L3	L1	L3	L2	L2	L1	L3	L1	L1	L3	L2
ANN18	L1	L2	L2	L2	L1	L2	L2	L1	L2	L1	L1	L3	L1	L2	L1	L3	L3	L2	L1	L2	L2	L1	L3
ANN19	L2	L1	L2	L2	L1	L1	L2	L2	L1	L2	L1	L1	L2	L1	L3	L3	L3	L1	L2	L2	L1	L2	L3
ANN20	L2	L1	L2	L2	L1	L1	L2	L2	L1	L2	L1	L2	L3	L2	L1	L1	L1	L2	L3	L3	L2	L3	L1
ANN21	L2	L1	L2	L2	L1	L1	L2	L2	L1	L2	L1	L3	L1	L3	L2	L2	L2	L3	L1	L1	L3	L1	L2
ANN22	L2	L1	L2	L1	L2	L2	L2	L1	L1	L1	L2	L1	L2	L2	L3	L3	L1	L2	L1	L1	L3	L3	L2
ANN23	L2	L1	L2	L1	L2	L2	L2	L1	L1	L1	L2	L2	L3	L3	L1	L1	L2	L3	L2	L2	L1	L1	L3
ANN24	L2	L1	L2	L1	L2	L2	L2	L1	L1	L1	L2	L3	L1	L1	L2	L2	L3	L1	L3	L3	L2	L2	L1
ANN25	L2	L1	L1	L2	L2	L2	L1	L2	L2	L1	L1	L1	L3	L2	L1	L2	L3	L3	L1	L3	L1	L2	L2
ANN26	L2	L1	L1	L2	L2	L2	L1	L2	L2	L1	L1	L2	L1	L3	L2	L3	L1	L1	L2	L1	L2	L3	L3
ANN27	L2	L1	L1	L2	L2	L2	L1	L2	L2	L1	L1	L3	L2	L1	L3	L1	L2	L2	L3	L2	L3	L1	L1
ANN28	L2	L2	L2	L1	L1	L1	L1	L2	L2	L1	L2	L1	L3	L2	L2	L2	L1	L1	L3	L2	L3	L1	L3
ANN29	L2	L2	L2	L1	L1	L1	L1	L2	L2	L1	L2	L2	L1	L3	L3	L3	L2	L2	L1	L3	L1	L2	L1
ANN30	L2	L2	L2	L1	L1	L1	L1	L2	L2	L1	L2	L3	L2	L1	L1	L1	L3	L3	L2	L1	L2	L3	L2
ANN31	L2	L2	L1	L2	L1	L2	L1	L1	L1	L2	L2	L1	L3	L3	L3	L2	L3	L2	L2	L1	L2	L1	L1
ANN32	L2	L2	L1	L2	L1	L2	L1	L1	L1	L2	L2	L2	L1	L1	L1	L3	L1	L3	L3	L2	L3	L2	L2
ANN33	L2	L2	L1	L2	L1	L2	L1	L1	L1	L2	L2	L3	L2	L2	L2	L1	L2	L1	L1	L3	L1	L3	L3
ANN34	L2	L2	L1	L1	L2	L1	L2	L1	L2	L2	L1	L1	L3	L1	L2	L3	L2	L3	L1	L2	L2	L3	L1
ANN35	L2	L2	L1	L1	L2	L1	L2	L1	L2	L2	L1	L2	L1	L2	L3	L1	L3	L1	L2	L3	L3	L1	L2
ANN36	L2	L2	L1	L1	L2	L1	L2	L1	L2	L2	L1	L3	L2	L3	L1	L2	L1	L2	L3	L1	L1	L2	L3

**Algorithm 1:**
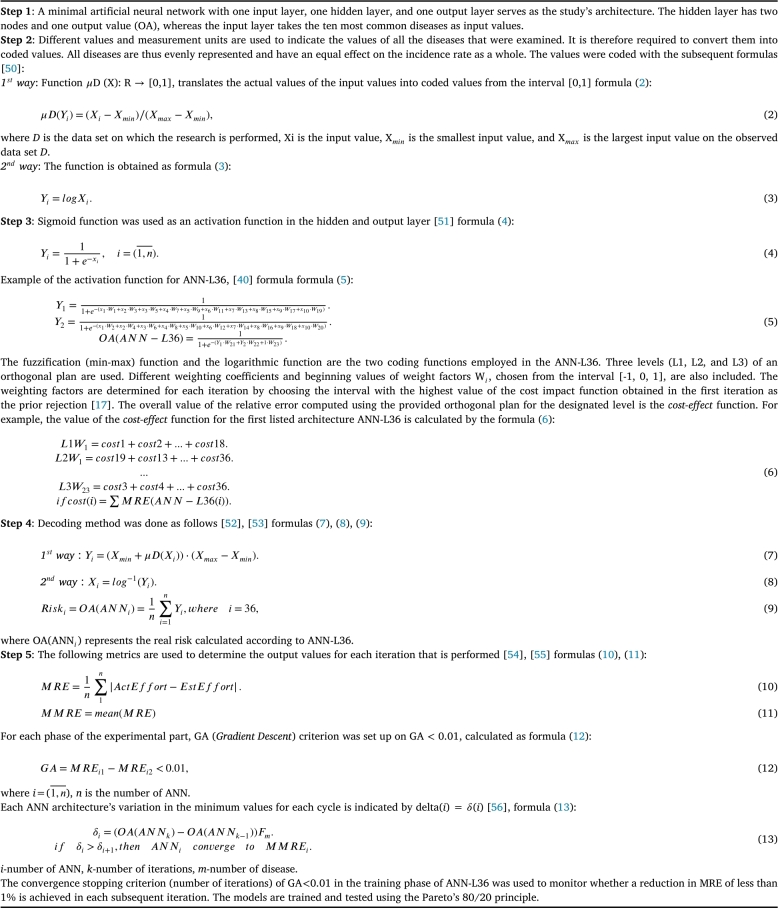
Robust design of the experiment [50],[51],[52],[53],[54],[55],[56].

### 3.3 Dataset description

A total of 27801 respondents from Serbia participated in the presented research, where 14623 (52.6%) respondents of both genders participated in the 2013 survey, and 13178 (47.4%) respondents of both genders participated in the 2019 survey. According to gender, 13190 (47.4%) were male respondents, and 14611 (52.6%) were female respondents. Observed by region: Belgrade region had 6760 (24.3%) respondents, Vojvodina region had 5925 (21.3%) respondents, Sumadija and Western Serbia region had 8659 (31.1%) respondents, and Eastern and Southern Serbia region had 6457 (23.2%) respondents. The respondents were of different age groups, from 15 to over 85 years old. Age groups' total share was as follows: from 15 to 24 years, 3241 (11.7%) respondents; from 25 to 34 years, 3590 (12.9%) respondents; from 35 to 44 years, 4140 (14.9%) respondents; from 45 to 54 years 4317 (15.5%) respondents; from 55 to 64 years 5268 (18.9%) respondents; from 65 to 74 years 4252 (15.3%) respondents; from 75 to 84 years 2510 (9.0%) respondents and over 85 years 483 (1.7%) respondents. The dataset consists of 17 chronic non-communicable diseases, where they are identified according to gender, region, and age groups for the 10 most prevalent diseases.

## 4 Results

**Figure 2:**
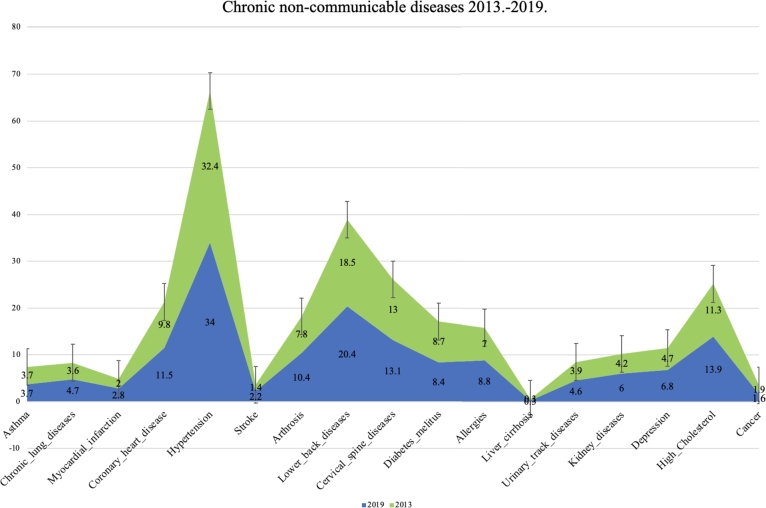
Seventeen chronic non-communicable diseases 2013 vs. 2019.

Within this section, we will showcase the outcomes derived from our study. It is evident from the findings that cardiovascular diseases encompass the most substantial portion of morbidity within the Serbian population. Hypertension is the most influenced in both periods, with 34.0% in 2013 and 33.2% in 2019. Lower back diseases are in second place, with 20.4% in 2013 and 19.55 in 2019. About half of the identified residents are affected by these two diseases, while the remaining 15 chronic diseases have the other half of the percentage share. The ranking goes as follows Cervical spine diseases with 13.1% in both years; High Cholesterol with 13.9% in 2013 and 12.6% in 2019; Coronary heart diseases with 11.5% in 2013 and 10.7% in 2019; Arthrosis with 10.4% in 2013 and 9.1% in 2019; Diabetes mellitus with 8.4% in 2013 and 8.5% in 2019; Allergies with 8.8% in 2013 and 8.0% in 2019; Depression with 6.8% in 2013 and 5.8% in 2019. The remaining chronic diseases are represented in a smaller percentage Fig. 2.

**Figure 3:**
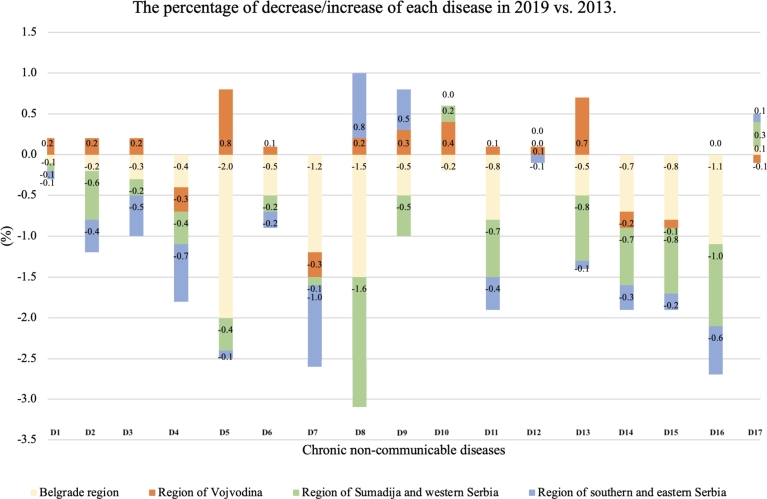
The percentage of the 17 chronic non-communicable diseases by region in 2019 compared to 2013.

The prevalence of chronic non-communicable diseases in Serbian population in 2013 and 2019 was estimated using a univariate regression model. Significant variations in the observed periods were found using a multivariate regression model. In 2013, observed according to four regions; statistically significant differences were identified in the following diseases: Chronic lung diseases, Coronary heart diseases, Hypertension, Stroke, Lower back diseases, Allergies, Urinary track diseases, and High Cholesterol. In 2019, observed according to four regions, significant differences were identified in the following diseases: Myocardial infarction, Hypertension, Arthrosis, Allergies, Depression, and High Cholesterol. Statistically significant differences in disease prevalence were observed between the two time periods, particularly among different age groups. The diseases showing significant differences include Coronary heart diseases, Hypertension, Arthrosis, Diabetes mellitus, Allergies, Kidney diseases, Depression, and High Cholesterol. It's worth noting that in almost all of these diseases, there was a significant decrease in incidence by region. The highest percentage share of all diseases is present among residents in the underdeveloped regions of Sumadija and western Serbia and the regions of eastern and southern Serbia, and significantly lower in the more developed regions of Belgrade and the region of Vojvodina Table 2. In Fig. 3 shows the percentage distribution of non-communicable chronic diseases by regions in 2019 as in contrast to 2013. Furthermore, we can draw the conclusion that the Sumadija and Western Serbia region saw the most percentage decline in Lower Back Diseases (1.6%). The Vojvodina region had the highest percentage increase in hypertension (0.8%), while the Southern and Eastern Serbia region had the highest percentage increase in lower back diseases (0.8%).

**Table 2:** Univariate and multivariate regression model of chronic non-communicable diseases in residents of Serbia in the period from 2013 to 2019 by region.

**CNCDs**	**Region**					
	2013			2019		
	(%)	OR(95% CI)	*p*	(%)	OR(95% CI)	*p*
Asthma	3.7	0.846	0.429	3.6	1.256	0.285
	0.974 *p*=0.404					
Chronic lung diseases	4.7	13.501	**0.000**	3.7	0.986	0.373
	1.690 *p*=0.167					
Myocardial infarction	2.8	0.240	0.787	2.0	9.955	**0.000**
	1.361 *p*=0.253					
Coronary heart disease	11.6	8.211	**0.000**	9.8	1.781	0.169
	3.636 ***p*=0.012**					
Hypertension	34.0	11.811	**0.000**	32.3	28.280	**0.000**
	15.122 ***p*=0.000**					
Stroke	2.2	4.538	**0.011**	1.4	0.599	0.549
	1.416 *p*=0.236					
Arthrosis	10.4	1.509	0.221	7.8	3.567	**0.028**
	2.820 ***p*=0.037**					
Lower back diseases	20.5	9.020	**0.000**	18.4	2.134	0.118
	2.098 *p*=0.0098					
Cervical spine diseases	13.2	0.775	0.461	13.0	1.456	0.233
	1.887 *p*=0.129					
Diabetes mellitus	8.4	0.927	0.396	8.8	0.446	0.640
	6.217 ***p*=0.000**					
Allergies	8.8	10.251	**0.000**	7.0	16.769	**0.000**
	9.668 ***p*=0.000**					
Liver cirrhosis	0.3	1.037	0.355	0.3	1.680	0.186
	1.037 *p*=0.375					
Urinary track diseases	4.6	6.301	**0.002**	3.9	2.356	0.095
	0.415 *p*=0.742					
Kidney diseases	6.1	1.661	0.190	4.2	1.562	0.210
	3.743 ***p*=0.011**					
Depression	6.7	1.033	0.356	4.8	7.263	**0.001**
	2.965 ***p*=0.031**					
High Cholesterol	13.9	30.564	**0.000**	11.2	12.818	**0.000**
	26.289 ***p*=0.000**					
Cancer	1.6	1.866	0.155	2.0	1.504	0.222
	1.256 *p*=0.288					

*Values marked bold represent statistically significant differences.

**OR(95% CI) represents Odds Ratio on 95% confidence interval.

**Figure 4:**
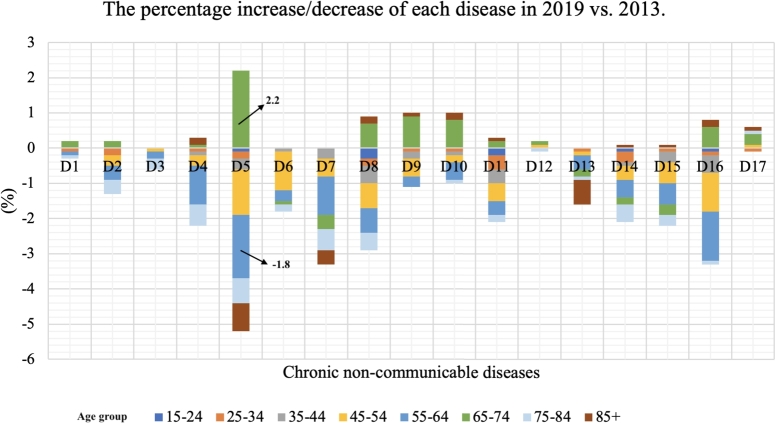
The percentage of the 17 chronic non-communicable diseases by age group in 2019 compared to 2013.

As expected, the results analyzed according to age groups show also statistically significant differences. In 2013, observed according to age groups, significant differences were identified in the following diseases: Chronic lung diseases, Coronary heart diseases, Hypertension, Stroke, Lower back diseases, Allergies, Urinary track diseases, and High Cholesterol. In 2019, observed according to age groups, significant differences were identified in the following diseases: Myocardial infarction, Hypertension, Arthrosis, Allergies, Depression and High Cholesterol. According to these two time periods concerning age groups, statistically, significant differences exist in almost all diseases, except for Stroke and Allergies. In almost all diseases, there was a significant decrease in incidence observed by age groups. The highest percentage share of all diseases is present in residents aged 64 to 75, the expected lowest in the age group 15 to 24 Table 3. Based on Fig. 4, it can be inferred that the age group of 55 to 64 exhibits the biggest percentage drop in hypertension (1.8%). Again, among those 65 to 74 years old, hypertension has seen the most increase (2.2%).

**Table 3:** Univariate and multivariate regression model of chronic non-communicable diseases in residents of Serbia in the period from 2013 to 2019 by age group.

**CNCDs**	**Age group**					
	2013			2019		
	(%)	OR(95% CI)	*p*	(%)	OR(95% CI)	*p*
Asthma	3.6	0.846	0.429	3.6	1.256	0.285
	3.697 ***p*=0.001**					
Chronic lung diseases	4.7	13.501	**0.000**	3.7	0.986	0.373
	2.456 ***p*=0.016**					
Myocardial infarction	2.7	0.240	0.787	2.0	9.955	**0.000**
	2.420 ***p*=0.018**					
Coronary heart disease	11.6	8.211	**0.000**	9.7	1.781	0.169
	5.194 ***p*=0.000**					
Hypertension	34.1	11.811	**0.000**	32.4	28.280	**0.000**
	2.813 ***p*=0.006**					
Stroke	2.3	4.538	**0.011**	1.5	0.599	0.549
	1.901 *p*=0.065					
Arthrosis	10.5	1.509	0.221	7.7	3.567	**0.028**
	2.637 ***p*=0.010**					
Lower back diseases	20.5	9.020	**0.000**	18.5	2.134	0.118
	2.146 ***p*=0.036**					
Cervical spine diseases	13.2	0.775	0.461	13.1	1.456	0.233
	2.158 ***p*=0.035**					
Diabetes mellitus	8.6	0.927	0.396	8.6	0.446	0.640
	4.181 ***p*=0.000**					
Allergies	8.8	10.251	**0.000**	7.0	16.769	**0.000**
	0.907 *p*=0.500					
Liver cirrhosis	0.2	1.037	0.355	0.3	1.680	0.186
	2.689 ***p*=0.009**					
Urinary track diseases	4.5	6.301	**0.002**	3.8	2.356	0.095
	2.962 ***p*=0.004**					
Kidney diseases	6.1	1.661	0.190	4.2	1.562	0.210
	3.031 ***p*=0.003**					
Depression	6.8	1.033	0.356	4.7	7.263	**0.001**
	3.100 ***p*=0.003**					
High Cholesterol	13.9	30.564	**0.000**	11.4	12.818	**0.000**
	11.043 ***p*=0.000**					
Cancer	1.5	1.866	0.155	2.0	1.504	0.222
	2.749 ***p*=0.007**					

*Values marked bold represent statistically significant differences.

**OR(95% CI) represents Odds Ratio on 95% confidence interval.

The obtained results analyzed according to gender, as expected, show statistically significant differences. In 2013, observed by gender, significant differences were identified in the following diseases: Chronic lung diseases, Coronary heart disease, Hypertension, Stroke, Lower back diseases, Allergies, Urinary track diseases, and High Cholesterol. In 2019, observed according to the gender, significant differences were identified in the following diseases: Myocardial infarction, Hypertension, Arthrosis, Allergies, Depression, and High Cholesterol. There are no statistically significant differences, except for the following diseases, observed according to these two time periods concerning gender: Coronary heart disease and Depression. In almost all diseases, there was a significant decrease in incidence observed by gender. A higher incidence of all diseases is present in the female part of the population compared to the male part observed in both periods Table 4.

**Table 4:** Univariate and multivariate regression model of chronic non-communicable diseases in residents of Serbia in the period from 2013 to 2019 by gender.

**CNCDs**	**Gender 2013-2019**					
	males			females		
	(%)	OR(95% CI)	*p*	(%)	OR(95% CI)	*p*
Asthma	1.7	0.846	0.429	2.1	1.256	0.285
	0.556 *p*=0.456					
Chronic lung diseases	1.8	13.501	**0.000**	2.4	0.986	0.373
	0.321 *p*=0.571					
Myocardial infarction	1.5	0.240	0.787	0.9	9.955	**0.000**
	1.591 *p*=0.207					
Coronary heart disease	4.3	8.211	**0.000**	6.4	1.781	0.169
	6.794 ***p*=0.009**					
Hypertension	13.9	11.811	**0.000**	19.3	28.280	**0.000**
	1.900 *p*=0.168					
Stroke	0.9	4.538	**0.011**	0.9	0.599	0.549
	0.031 *p*=0.859					
Arthrosis	2.6	1.509	0.221	6.5	3.567	**0.028**
	0.538 *p*=0.463					
Lower back diseases	7.3	9.020	**0.000**	12.2	2.134	0.118
	2.908 ***p*=0.088**					
Cervical spine diseases	4.1	0.775	0.461	9.0	1.456	0.233
	1.276 *p*=0.259					
Diabetes mellitus	3.9	0.927	0.396	4.7	0.446	0.640
	0.195 *p*=0.659					
Allergies	2.8	10.251	**0.000**	5.2	16.769	**0.000**
	1.905 *p*=0.168					
Liver cirrhosis	0.2	1.037	0.355	0.2	1.680	0.186
	1.094 *p*=0.296					
Urinary track diseases	2.1	6.301	**0.002**	2.2	2.356	0.095
	0.052 *p*=0.819					
Kidney diseases	2.0	1.661	0.190	3.1	1.562	0.210
	1.262 *p*=0.261					
Depression	1.9	1.033	0.356	3.8	7.263	**0.001**
	5.051 ***p*=0.025**					
High Cholesterol	4.8	30.564	**0.000**	7.9	12.818	**0.000**
	0.512 *p*=0.474					
Cancer	0.6	1.866	0.155	1.1	1.504	0.222
	2.216 *p*=0.137					

*Values marked bold represent statistically significant differences.

**OR(95% CI) represents Odds Ratio on 95% confidence interval.

**Figure 5:**
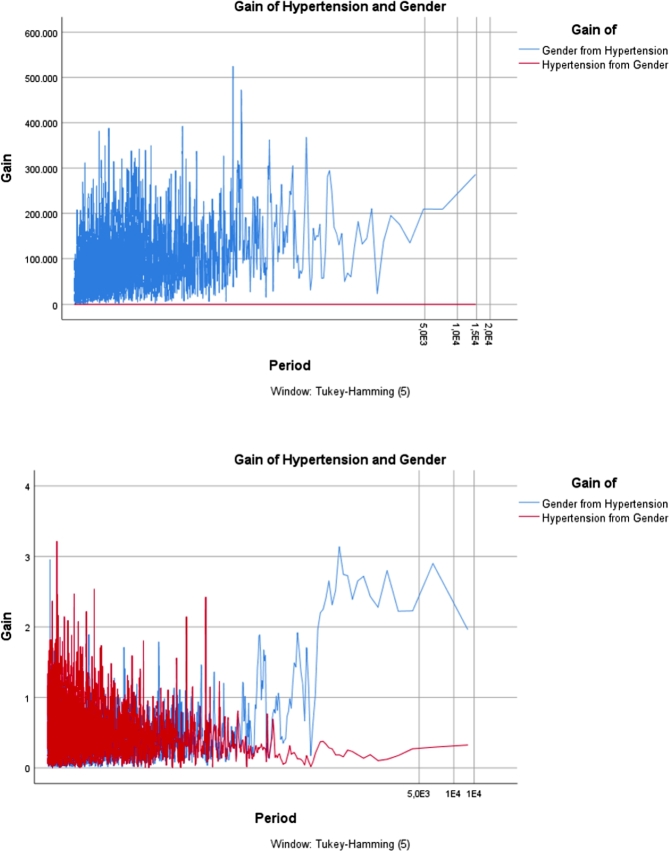
*Gain* of Gender for Hypertension 2013 Vs. 2019.

**Figure 6:**
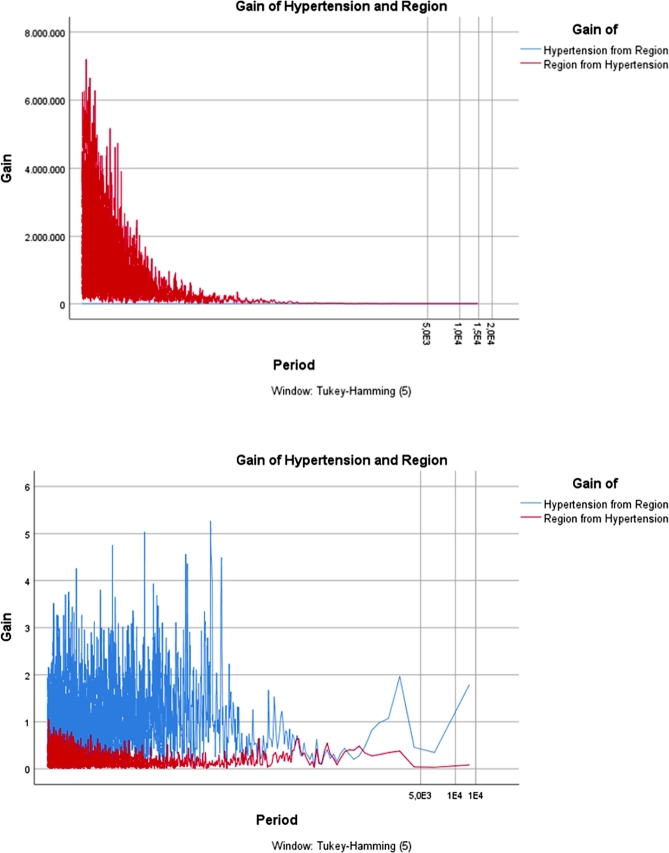
*Gain* of Region for Hypertension 2013 Vs. 2019.

**Figure 7:**
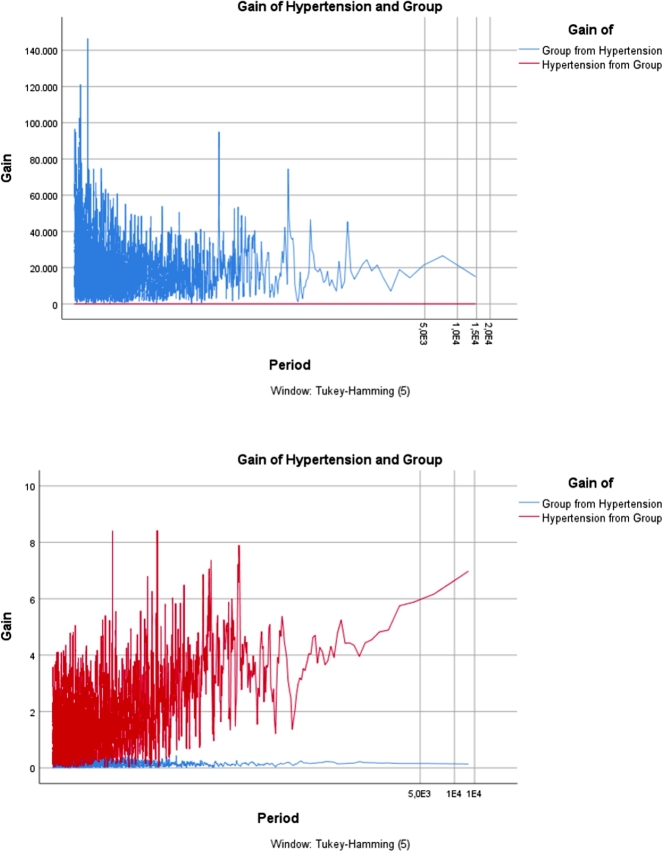
*Gain* of Group for Hypertension 2013 Vs. 2019.

The *“gain ratio”* is primarily used in the field of machine learning, specifically in decision tree algorithms for attribute selection. It is employed to determine the relevance of a feature (attribute) in a dataset. Gain ratio is an improvement over information gain, addressing the bias that information gain has towards attributes with a larger number of distinct values. It's often used to help decision trees make more informed splits by considering the intrinsic characteristics of the attributes. In order to investigate the incidence rate of chronic non-communicable diseases, particularly focusing on the prominent ailment of Hypertension, across various factors including region, age group, and gender, the graphical depiction of the gain ratio is presented in Figure 5, Figure 6, Figure 7. Furthermore, these graphical illustrations of gain ratios across various regions, age groups, and genders provide a comprehensive overview of the data distribution. This use of the gain ratio allows for a thorough exploration of the examined disease's incidence rate.

Table 5 shows the average incidence rate values for both years using two different coding functions. The convergence speed in all 4 cases is extraordinarily high, and less than 8 iterations are needed to reach the value of the lowest relative error. The GA criterion <0.01 is met in each subsequent iteration.

**Table 5:** Number of performed iterations - ANN-L36-Fuzzy vs. ANN-L36-Log.

**ANN-L36**	1.iteration	2.iteration	3.iteration	4.iteration	5.iteration	6.iteration	7.iteration
2013-F	66.3	37.4	26.5	19.7	15.2	13.8	13.3
2019-F	59.8	35.3	23.7	18.9	15.6	13.4	12.6
2013-L	69.4	38.6	28.9	20.1	16.1	13.9	13.4
2019-L	56.7	31.1	22.8	17.3	15.4	13.2	12.7

Table 6 shows the MMRE values for all used models in both training and testing phases, observed on two different dates for 2013 and 2019. The used Decision Tree, observed by gender, region, and age group, gives the lowest value of MMRE in the training and testing phase for gender in 2019, while it achieves the highest value of MMRE by age group for 2013. The SVR algorithm uses two different kernel functions, where the lowest MMRE value is achieved in the training phase for 2013, observed concerning gender, age, and region. By using ANN RBF-N-RE, the lowest MMRE value of 0.2% and 0.1% for 2013 and 0.5% and 0.1% for 2019 is achieved in the training and testing phases, respectively. Using two new proposed models, ANN-L36-Fuzzy and ANN-L36-Log, a better result, i.e., the lowest value of MMRE, can be observed with the first model. In the training and testing phase for 2013, the MMRE value is 0.1% and 0.2%, respectively, while in the training and testing phases for 2019, the MMRE value is 0.1% and 0.1%.

**Table 6:** MMRE(%) for all models used.

**Model**	**2013-MMRE(%)**		**2019-MMRE(%)**	
	Training	Testing	Training	Testing
Decision Tree GE	0.4	0.8	0.4	0.4
Decision Tree RE	0.8	0.8	0.6	0.6
Decision Tree AG	2.6	2.6	0.5	0.5
SVR-LNR	1.8	2.1	1.9	2.2
SVR-POLY	1.3	1.5	1.7	1.8
ANN-RBF-S-GE	0.4	0.3	0.3	0.3
ANN-RBF-S-RE	0.2	0.3	0.3	0.1
ANN-RBF-S-AG	5.4	4.0	3.3	3.0
ANN-RBF-N-GE	0.5	0.4	0.5	0.3
ANN-RBF-N-RE	0.2	0.1	0.5	0.1
ANN-RBF-N-AG	4.3	4.1	9.5	8.5
ANN-L36-Fuzzy	**0.1**	**0.2**	**0.1**	**0.1**
ANN-L36-Log	0.2	0.4	0.3	0.5

**Figure 8:**
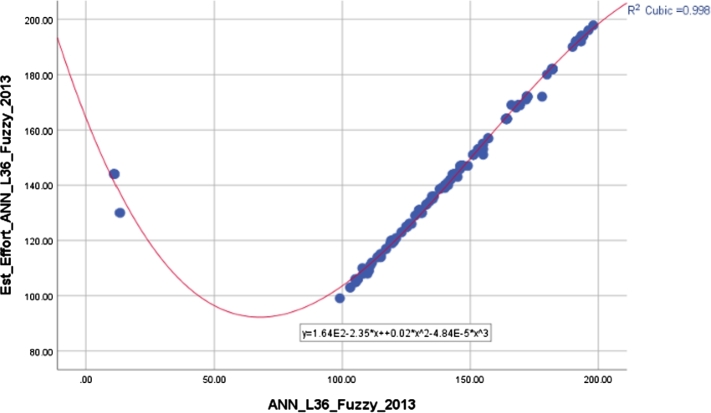
R^2^ cubic for ANN-L36-Fuzzy observed for 2013.

**Figure 9:**
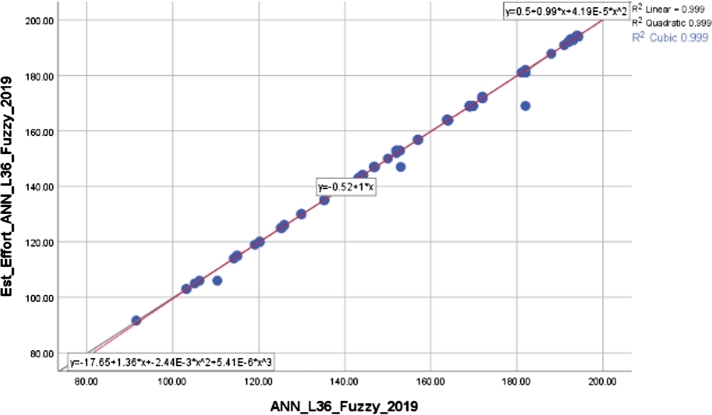
R^2^ cubic for ANN-L36-Fuzzy observed for 2019.

The robustness of the proposed methods and constructed models refers to their resistance or stability. Many inferences can be made from the correlation coefficients between the estimated and actual values in 2013 and 2019, which are shown in Table 7. On the one hand, the ANN-RBF-N-AG model, which was built using gender-based analysis and normalization of the Radial Basic Function, has the lowest correlation. On the other hand, the ANN-L36-Fuzzy architecture, utilizing an ANN design constructed through Taguchi's orthogonal plan with minimal iterations and employing a fuzzy function for input variables, demonstrates the highest accuracy. The ML algorithms, including Decision Tree and SVR, when comparing different participant categories, achieve a high level of accuracy and precision ranging from 0.6 to 0.8. However, the ANN-L36 architecture achieves an exceptional level of accuracy and precision exceeding 0.9. Graphical representations for the best model, ANN-L36-Fuzzy, illustrating the relationship between estimated and real values for the years 2013 and 2019, are provided in Fig. 8 and Fig. 9. Finally, the X-axis represents the actual percentage value, while Est_Effort is the obtained value using the proposed ANN-L36-Fuzzy model. The deterministic coefficient R^2^ robustly elucidates the correlation between the actual and estimated values.

**Table 7:** Correlation coefficients 2013-2019.

Models	Pearson 2013-2019	Spearman's rho 2013-2019	R^2^ cubic 2013	R^2^ cubic 2019
Decision Tree GE	0.765	0.783	0.771	0.778
Decision Tree RE	0.735	0.751	0.745	0.749
Decision Tree AG	0.678	0.775	0.690	0.721
SVR-LNR	0.754	0.773	0.765	0.768
SVR-POLY	0.767	0.782	0.771	0.779
ANN-RBF-S-GE	0.753	0.781	0.763	0.769
ANN-RBF-S-RE	0.791	0.833	0.801	0.814
ANN-RBF-S-AG	0.677	0.692	0.673	0.685
ANN-RBF-N-GE	0.713	0.733	0.722	0.729
ANN-RBF-N-RE	0.782	0.813	0.794	0.805
ANN-RBF-N-AG	0.643	0.678	0.653	0.662
ANN-L36-Fuzzy	**0.982**	**0.991**	**0.998**	**0.999**
ANN-L36-Log	0.976	0.984	0.979	0.981

## 5 Discussion

Each region has a variable prevalence of chronic non-communicable diseases, which is inversely correlated with the region's level of development. The most prosperous regions of Belgrade, with an average of 2.4%, and Vojvodina, with 1.9%, have the lowest incidence rate of chronic non-communicable diseases, of which cardiovascular diseases lead with hypertension as the leading disease. In contrast to these two regions, the less developed regions of Sumadija and western Serbia, with an average of 2.6%, and the region of eastern and southern Serbia, with an average of 2.2%, have a higher incidence rate, but still with leading cardiovascular diseases. Observed by age groups, the research showed that the most vulnerable age groups with the highest incidence rate are from 75 to 84 years old, followed by the age group from 65 to 74. On the other hand, the respondents over 85 years old had a significantly lower percentage of disease on average in 2013, around 9.0%, while in 2019, it was 7.9%. As expected, the lowest percentage of the disease is in the age group of young people from 15 to 24 years old. All analyzes conclude that the female population gets sick significantly more than the male population in Serbia. In almost all chronic non-communicable diseases, the percentage of female respondents was 10.5% in 2013, and 4.6% in 2019 is significantly higher than that of male respondents, an average of 7.3% in 2013 and 3.3% in 2019. Based on the obtained results, the set research objectives of this study can be answered in the following way:

***RO1***: Introducing the new methodology with two coding functions for the ANN-L36 model for predicting the increase/decrease or constant spread of chronic diseases in the period from 2013 to 2019.

All models showed that the incidence rate of almost all non-communicable diseases in Serbia is in decline and the percentage share of each individual disease is significantly lower in 2019 compared to 2013. Using the ANN-L36 model, it was additionally established that the incidence rate in the highly developed regions of Belgrade and Vojvodina is significantly lower than in the less developed regions of Sumadia and western Serbia, as well as eastern and southern Serbia. It was also determined that the percentage share of each chronic non-communicable disease is significantly higher in both observed periods (2013 and 2019). As expected, there are significant differences in the incidence rate according to age group, where the percentage of each disease is highest among the population aged 65 to 74 and the lowest among the population aged 15 to 24. It can be concluded that in the following period, the percentage share of each of the observed diseases will gradually decrease.

***RO2***: Comparison of the new ANN-L36 model with ML algorithms and other artificial neural networks based on different hidden layer functions.

By comparing the MMRE values for each of the used models, it can be concluded that ANN-L36 is the model with the slightest error that can precisely and accurately determine the incidence rate of chronic non-communicable diseases.

## 6 Conclusion

There haven't been any studies that provide data on all seventeen chronic non-communicable diseases in Serbia at the national level, based on what is currently known and the literature that is available. In developing countries like Serbia, the impact of cardiovascular diseases is the most prevalent, often leading to fatal outcomes such as heart attacks. For these reasons, it is imperative to change lifestyles, undergo preventive screenings, and improve the entire healthcare system. Prior to 2013, research indicated an increase in the number of chronic non-communicable diseases among the population in Serbia. However, this study demonstrates that overall healthcare system improvement can lead to better health outcomes for the entire society. As a result, a proportional reduction in the incidence of almost every chronic non-communicable disease in Serbia was observed between 2013 and 2019. These findings highlight the positive effect of organized preventive screenings, which need to be intensified and expanded to areas and age groups with higher disease prevalence. For instance, the most prevalent disease in Serbia, hypertension, showed a slight decrease from 34.0% to 32.2% during the observed period.

Furthermore, the proposed models for regional and age group analysis can significantly contribute to the preventive healthcare system. These models enable rapid and efficient analysis of a large amount of collected data and identification of the most vulnerable regions, age groups, and genders. The most accurate model was the ANN-L36 Fuzzy algorithm with a relative error of 0.1% and correlation coefficients exceeding 0.98. Their application is not limited to medicine but can be utilized in various scientific fields such as neuroscience, pattern recognition, nuclear science, robotics, and others. With the continuous advancements in artificial intelligence diagnostics, the proposed approach can be used to develop tools that provide precise and rapid assessment of functionality necessary for diagnosing and treating different diseases. This can greatly assist professionals and medical teams in overcoming challenges frequently encountered in the field of medical diagnostics. By employing ML algorithms and ANN architecture, our proposed approach offers an efficient and effective solution for identifying the most vulnerable regions and age groups affected by diseases, ultimately leading to improved patient outcomes.

Finally, comprehensive efforts to monitor and identify chronic diseases in the way suggested by this study have not been carried out on a national scale in Serbia. The model introduced in this research is poised for practical implementation, offering a substantial contribution to Serbia's forthcoming healthcare framework. This contribution will play a pivotal role in refining and advancing the approach to tackling these conditions. Moreover, empirical evidence has compellingly demonstrated that Taguchi's optimization approach outperforms other methods in pinpointing a range of chronic non-communicable diseases.

### 6.1 Limitations and recommendations for future research

The limits of missing data must be taken into account when applying Decision Trees, Support Vector Regressors, and Artificial Neural Networks to forecast chronic non-communicable diseases based on national medical datasets. The dataset's missing values have the potential to generate biases and reduce the predictive models' accuracy. Incomplete patient records, resulting from missing data, can impede the comprehensive analysis and prediction process, as these models rely on complete data for accurate predictions.

Furthermore, missing data can lead to reduced sample sizes, compromising the statistical power of predictive models. Models' ability to identify meaningful relationships and patterns linked to chronic non-communicable diseases may be hampered by smaller sample sizes, which would restrict the applicability of their conclusions to the whole population. Additionally, imputing missing values introduces challenges, as the chosen imputation methods may introduce assumptions and uncertainties that impact the accuracy and reliability of predicted outcomes.

In light of these drawbacks, deeper learning models should be the main focus of future studies that use various national medical datasets to forecast chronic non-communicable diseases. Recurrent neural networks (RNNs), one type of deep learning model, have demonstrated promise in handling missing data and discovering intricate patterns from large-scale datasets. Particularly, some RNN types, such as Fuzzy Cognitive Maps, may carry out different *WHAT-IF* simulations and look for hidden patterns in the data. By leveraging the capabilities of deep learning models, researchers can potentially address the challenges associated with missing data, enhancing the accuracy, reliability, and generalizability of predictive models for chronic non-communicable diseases.

**Declarations**

## Funding

No funds, grants, or other support were received.

## Ethics approval

Research Ethics Committee from the Institute of the Public health “Dr. Milan Jovanovic - Batut”, protocol code: 7959/1. Data and/or Code availability: The raw dataset used for this study is under a Non-Disclosure Agreement (NDA) and is, therefore, unavailable to the public. All patients participated in the national study via random sampling, voluntarily and anonymously, and their consent was obtained. Personal data related to study participants is not available.

## CRediT authorship contribution statement

**Nevena Rankovic:** Writing – review & editing, Writing – original draft, Visualization, Validation, Software, Methodology, Investigation, Formal analysis, Conceptualization. **Dragica Rankovic:** Writing – review & editing, Writing – original draft, Visualization, Supervision, Methodology, Investigation, Formal analysis, Conceptualization. **Igor Lukic:** Writing – original draft, Resources, Investigation, Formal analysis, Data curation. **Nikola Savic:** Writing – review & editing, Validation, Resources, Investigation, Data curation. **Verica Jovanovic:** Writing – review & editing, Validation, Resources, Data curation.

## Declaration of Competing Interest

The authors declare that they have no known competing financial interests or personal relationships that could have appeared to influence the work reported in this paper.

## Data Availability

The authors do not have the permission to share the data.
